# Contribution of OqxAB Efflux Pump in Selection of Fluoroquinolone-Resistant *Klebsiella pneumoniae*

**DOI:** 10.1155/2018/4271638

**Published:** 2018-09-23

**Authors:** Orsolya Szabo, Bela Kocsis, Nikolett Szabo, Katalin Kristof, Dora Szabo

**Affiliations:** ^1^Institute of Medical Microbiology, Semmelweis University, Budapest 1082, Hungary; ^2^Institute of Laboratory Medicine, Clinical Microbiology Laboratory, Semmelweis University, Budapest 1089, Hungary

## Abstract

The role of OqxAB efflux pump in *Klebsiella pneumoniae* was investigated in correlation with ciprofloxacin exposure. *K. pneumoniae* SE23 and *K. pneumoniae* SE191 were isolated from urinary tract infections and were analyzed in this study. Each carried *oqxAB* resistance determinant and exhibited ciprofloxacin MIC of 0.06 and 0.5 mg/L, respectively. Tested strains were initially exposed to their ciprofloxacin MIC values for 24 hours. Later on, the ciprofloxacin exposition has been increased to a daily 1, 2, 4, and to a final 8 mg/L. Total cellular RNA was extracted at 30, 60, 90, and 120 minutes of initial exposure and after every 24 hours. Quantitative reverse-transcriptase PCR was performed from each RNA sample. Mutation in *gyrA* and *parC* genes was analyzed in each strain and multilocus sequence typing (MLST) was performed. Ciprofloxacin exposure selected resistant strain from *K. pneumoniae* SE191; by contrast, *K. pneumoniae* SE23 was not adjustable to the increasing ciprofloxacin concentrations. During initial exposure, both *oqxA* and *oqxB* expression remained low (2^−ΔCt^ = 1-2.03). However, increasing ciprofloxacin promoted *oqxB* expression as it reached fold increase of 15.8–22.8, while *oqxA* expression was maintained (2^−ΔCt^ = 2-2.15). An amino acid substitution Ser83Tyr in *gyrA* was detected in *K. pneumoniae* SE191, but no additional mutations occurred as consequence to ciprofloxacin exposure. MLST identified *K. pneumoniae* SE191 as ST274, while *K. pneumoniae* SE23 belonged to the novel ST2567. Ciprofloxacin concentration-dependent upregulation of *oqxAB* efflux pump in *K. pneumoniae* is clonally related and contributes to selection for higher level of fluoroquinolone resistance.

## 1. Introduction

*Klebsiella pneumoniae* is one of the most widespread nosocomial pathogen and responsible for various infectious diseases, including urinary tract, bloodstream, and respiratory tract infections. It is capable of developing resistance to cephalosporins, carbapenems, fluoroquinolones, aminoglycosides, and recently to polymyxins [1–3]. Fluoroquinolone resistance in *K. pneumoniae* is explained by mutations in gyrase and topoisomerase IV enzymes; however, reduced susceptibility to fluoroquinolones and low-level resistance is maintained by plasmid-mediated quinolone resistance (PMQR) determinants, namely, Qnr determinants, QepA and OqxAB efflux pumps, and aminoglycoside-acetyltransferase Ib-cr enzyme [4].

OqxAB efflux pump belongs to the resistance nodulation division family and it is constructed by two main domains, namely, OqxA, a periplasmic part, and OqxB, a transmembrane protein. Its efflux pump activity is described in a TolC-independent way; however, presence of an outer membrane protein enhances its effect [5]. OqxAB has been commonly found chromosomally in *K. pneumoniae* and usually plasmid located in other Enterobacteriaceae species. This resistance determinant is responsible to develop reduced susceptibility and even resistance to olaquindox and other fluoroquinolone agents such as ciprofloxacin, norfloxacin, and flumequine [5, 6]. High prevalence of OqxAB has been described in *K. pneumoniae* on both chromosome and plasmids representing a potential reservoir of this resistance determinant [7–10]. In *K. pneumoniae*, upregulation of *oqxAB* efflux pump is *rarA* mediated; by contrast, *oqxR* is capable of downregulating it [11–13]. It has been recently reported that AcrAB associated with overexpression of OqxAB is required for high virulence potential in *K. pneumoniae* [14].

Internationally successful high-risk clones of *K. pneumoniae* have disseminated in Hungary. These clones were fluoroquinolone resistant and harboured extended-spectrum beta-lactamases (*bla*_CTX-M-15_) or carbapenemases (*bla*_VIM-4_, *bla*_KPC_); however, they retained fitness and these combined features contributed to the widespread dissemination of ST11, ST15, ST147, and ST258 in hospital settings and made them responsible for the majority of healthcare-associated infections [15–17]. Recently, ST274 has emerged in adult and newborn hospital wards and has been reported in several nosocomial infections [17]. The aim of this study was to analyze role of *oqxAB* efflux pump in selection of fluoroquinolone resistance in different *K. pneumoniae* clones.

## 2. Materials and Methods

### 2.1. Bacterial Strains

Two *K. pneumoniae* strains (*K. pneumoniae* SE23 and *K. pneumoniae* SE191) have been included in this study. Both have been previously found positive to *oqxAB* resistance determinant by PCR and nucleic acid sequencing and lacked all other PMQRs. Antimicrobial susceptibility testing was performed by microdilution method based on documents of the European Committee on Antimicrobial Susceptibility Testing (EUCAST), and both *K. pneumoniae* SE23 and *K. pneumoniae* SE191 were found susceptible to ciprofloxacin with MIC 0.06 and 0.5 mg/L, respectively, based on EUCAST breakpoints issued in the year 2016. However, ciprofloxacin resistance breakpoint of Enterobacteriaceae has been revised by EUCAST in 2017, indicating 0.5 mg/L value as nonsusceptible to fluoroquinolones.

### 2.2. Exposure to Ciprofloxacin

Each tested strain has been prepared in 0.5 McFarland density in Mueller-Hinton broth and has been initially exposed to their ciprofloxacin MIC values of 0.06 and 0.5 mg/L for 24 hours at 37°C. RNA extraction has been carried out at 30, 60, 90, and 120 minutes and at 24 hours of ciprofloxacin exposition. Later on, after the initial incubation, each tested strain has been adjusted to 0.5 McFarland density and has been exposed to an increased ciprofloxacin concentration. In the case of *K. pneumoniae* SE23, the peak ciprofloxacin exposition was 0.5 mg/L. On the other hand, *K. pneumoniae* SE191 was capable of adapting to a daily increasing 1, 2, 4, and 8 mg/L ciprofloxacin exposition, where each incubation at 37°C lasted for 24 hours. After each daily exposure, RNA extraction has been performed from each sample to analyze expression of *oqxAB* efflux pump.

### 2.3. Quantitative Reverse-Transcriptase PCR Analysis for Expression of oqxAB

Total cellular RNA was extracted from both tested strains at given time point of incubation by RNeasy Mini Kit (Qiagen GmbH, Hilden, Germany) according to the manufacturer's instructions. The quantitative RT-PCR was carried out in a Step One Real-Time PCR System (Applied Biosystems, Thermo Fisher Scientific) with following thermal profile: 60°C for 30 sec; 50°C for 5 min and 95°C for 10 min; 40 cycles of 95°C for 15 sec and 60°C for 1 min; and 60°C for 30 sec.

To test separate expression of the *oqxA* and *oqxB* gene, the internal fragments were used to design set of primers and 6-FAM- or VIC-labelled probes. Chromosomal *rpoB* was chosen as housekeeping gene. All oligonucleotide primers and probes for quantitative RT-PCR were designed by Primer Express 3.0 software and are listed in Table 1. Each sample was tested in triplicate. The Ct values of genes of interest were normalized (ΔCt) to the Ct values of housekeeping gene *rpoB*, and the relative expression of each gene of interest was calculated as 2^−ΔCt^.

### 2.4. Analysis of Mutations in *gyrA* and *parC* Genes

Mutations in *gyrA* and *parC* genes have been investigated by PCR and nucleic acid sequencing. Set of primers was designed by online tools of MWG Eurofins Operon and synthetised by IDT Bioscience. PCR thermal profile was as follows: initial denaturation at 95°C for 3 min, 30 cycles of 95°C for 1 min, 52°C for 1 min, 72°C for 1 min, and an additional extension at 72°C for 5 min.

### 2.5. Multilocus Sequence Typing (MLST) of *K. pneumoniae*

Each tested strain has been analyzed by multilocus sequence typing based on sequences of seven housekeeping genes, namely, *gapA, infB, mdh, pgi, phoE, rpoB,* and *tonB*. Oligonucleotide primers and PCR thermal profiles were applied after Diancourt et al. article [18] and as it is highlighted on MLST database website http://bigsdb.pasteur.fr/klebsiella/primers_used.html.

Amplicons of MLST, *gyrA* and *parC* PCR were analyzed by electrophoresis in a 1.5% agarose gel (Sigma-Aldrich) at 120 V for 20 min in 1xTAE (40 mM Tris-HCl (pH 8.3), 2 mM acetate, and 1 mM EDTA), and gel was stained with 0.05 mg/L GelRed dye (Biotium) and visualised on a UV transilluminator. PCR-positive amplicons were purified by QIAquick PCR Purification Kit (Qiagen GmbH, Hilden, Germany), and nucleic acid sequencing was performed by BIOMI Kft. Gödöllő, Hungary.

### 2.6. Statistical Analysis

Expression level differences were analyzed by *t*-test for 2 independent means.

## 3. Results

*K. pneumoniae* SE23 and *K. pneumoniae* SE191 strains were positive to *oqxAB* by PCR and were both initially exposed to ciprofloxacin concentration of their MIC values 0.06 and 0.5 mg/L, respectively. However, selection of resistant strains was successful only by *K. pneumoniae* SE191 and it was found to be adjustable for further ciprofloxacin exposition. Hence, multiple conditions *K. pneumoniae* SE23 was not capable of adapting to increased ciprofloxacin concentrations. The highest ciprofloxacin value was 0.5 mg/L, where RNA could have been extracted from *K. pneumoniae* SE23, but no further exposition was possible.

**Figure 1:**
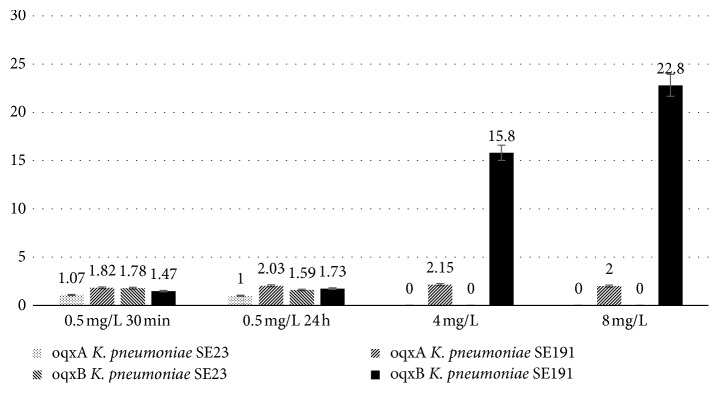
Expression in SE23 and SE191.

Quantitative RT-PCR was performed on RNA samples taken at given time points of ciprofloxacin exposure to investigate expression level differences of *oqxAB* efflux pump. Expression in SE191 to 0.5 mg/L for 30 minutes was 1.82 and no relevant expression change to 4 and 8 mg/L as 2^−ΔCt^ values were 2.15 and 2.00. In the case of SE23 expression, 2^−ΔCt^ values were 1.07 and 1 at maximum 0.5 mg/L (Figure 1).

In the case of OqxAB, expression level values calculated as 2^−ΔCT^ were 1.47 and 1.73 for SE191 while in SE23 values were 1.78 and 1.59. The change in expression of SE191 to 4 and 8 mg/L concentrations was in time 15.8 and 22.8 (Figure 1). Expression analysis by *t*-test showed *t* value = 1.88 and *p* value = 0.04.

In the case of SE191, a Ser83Tyr mutation in gyrase A has been detected. This mutation presented the 0.5 mg/L MIC value. In SE23, amino acid substitutions have not been at 0.5 mg/L exposition.

MLST has been conducted on both strains. *K. pneumoniae* SE191 was identified as ST274, while *K. pneumoniae* SE23 presented the novel ST2567. This novel sequence type incorporated a new *tonB* variant 371, which had two mutations at C48A and G61C compared to its closest match allele 87. Other alleles of *K. pneumoniae* SE23 yielded *gapA* 4, *infB* 31, *mdh* 13, *pgi* 1, *phoE* 1, and *rpoB* 1. The nucleic acid sequence of novel *tonB* allele 371 and the MLST profile of ST2567 have been submitted to *K. pneumoniae* database (http://bigsdb.pasteur.fr/klebsiella/klebsiella.html).

## 4. Discussion

Our study demonstrated role of OqxAB efflux pump in *K. pneumoniae* during exposure to increased ciprofloxacin concentrations. *K. pneumoniae* SE23 and *K. pneumoniae* SE191 belonging to different sequence types ST2567 and ST274, respectively, have been investigated. They showed varying ability to adapt to ciprofloxacin exposition, though both strains carried *oqxAB* efflux pump. The two tested strains exhibited different ciprofloxacin MIC values 0.06 and 0.5 mg/L, respectively, and both were susceptible based on the EUCAST 2016 documents (valid at the time when this experiment was started). The initial exposure selected resistant strains only in the ST274 clone and it was further exposed to higher ciprofloxacin concentrations untill 8 mg/L. This strain harboured already a Ser83Tyr amino acid substitution in gyrase A subunit, but no other mutations occurred as consequence to ciprofloxacin exposure. An upregulation of *oqxB* has been detected with a fold change of 22.8 times increase in expression during the ciprofloxacin exposure from 0.5 till 8 mg/L. This upregulation and selection of resistant strains seem clonally dependent as *K. pneumoniae* SE23, a strain of ST2567, could not increase the expression level of *oqxAB* and failed to adapt to an increasing ciprofloxacin concentration. This upregulation of efflux pump can contribute to the fitness of ST274, as it has been identified as one of the disseminated clones in newborn and adult hospital wards in Hungary [17].

Results of our study are in accordance with recent ciprofloxacin breakpoint modification of EUCAST. Our study had begun in 2016; therefore, antimicrobial susceptibility testing and interpretation of results were based on EUCAST 2016 documents where 0.5 mg/L ciprofloxacin value was in susceptible range. The resistance breakpoint of Enterobacteriaceae has been revised in 2017 and set 0.5 mg/L in nonsusceptible range. *K. pneumoniae* SE191 exhibiting 0.5 mg/L ciprofloxacin MIC was capable of developing further fluoroquinolone resistance by upregulation of *oqxAB* efflux pump due to ciprofloxacin exposure. By contrast, *K. pneumoniae* SE23 exhibiting 0.06 mg/L ciprofloxacin MIC and lacking increased *oqxAB* expression during exposure to ciprofloxacin was not able to adapt to fluoroquinolone exposition and could not develop resistance.

In our study, a novel *K. pneumoniae* sequence type has been detected, namely, ST2567 that was distinguished by two mutations in *tonB* allele from its closest sequence type 2387. The new *K. pneumoniae* ST profile has been deposited to MLST database (http://bigsdb.pasteur.fr/klebsiella/klebsiella.html).

Earlier studies demonstrated OqxAB efflux pump in extended-spectrum beta-lactamase-producing and fluoroquinolone-resistant *K. pneumoniae* high-risk clones [19–21]. In Hungary, *K. pneumoniae* with *oqxAB* resistance determinant was reported in bloodstream infections [22]. In all these reported studies, tested strains were resistant to fluoroquinolones. In our study, we demonstrated the role of *oqxAB* efflux pump in *K. pneumoniae* in selection of resistance to fluoroquinolones. Our two tested strains were susceptible to fluoroquinolones, but after exposure to ciprofloxacin, the one with *gyrA* mutation could be selected to higher levels of fluoroquinolone resistance. Additional chromosomal mutations of *gyrA* and *parC* genes in tested strain did not occur, suggesting that the development of resistance during exposure to 0.5–8 mg/L ciprofloxacin was mainly caused by the increased expression of the *oqxAB* efflux pump gene. Strains were exposed to the maximum of 8 mg/L ciprofloxacin concentration, and in human tissues, ciprofloxacin is unable to reach higher concentrations during a *per os* dosing; thus, the concentrations used in our study can represent a fluoroquinolone exposure during *per os* therapy.

## Figures and Tables

**Table 1:** Oligonucleotide primers and probes of this study.

Primer or probe	Sequence	Reference
gyrA fwd	CAGCCCTTCAATGCTGATG	Designed in the study
gyrA rev	CGCTTTTACTCCTTTTCTGTTC	Designed in the study
parC fwd	CTCAATCAGCGTAATCGCC	Designed in the study
parC rev	AATCCTCAGCCGATCTCAC	Designed in the study
Kpn.rpoBF1 fwd	GTCGCGGCTGAACAAGCT	Designed in the study
Kpn.rpoBR1 rev	AACGGCCACTTCGTAGAAGATC	Designed in the study
Kpn.rpoBP1-VIC probe	CTACGGCAGGTAACC	Designed in the study
oqxAF1 fwd	GTCGACGGCTTACAAAAAGTGTT	Designed in the study
oqxAR1 rev	GCAACGGTTTTGGCGTTAA	Designed in the study
oqxAP1-FAM probe	ATGCCGGGTATGCC	Designed in the study
oqxBF1 fwd	CTGGATTTTCCGTCCGTTTAAC	Designed in the study
oqxBR1 rev	TTGCCTACCAGTCCCTGATAGC	Designed in the study
oqxBP1-FAM probe	CTGCGCAGCTCGAA	Designed in the study

## Data Availability

Novel *K. pneumoniae* MLST profile of ST2567 and new *tonB* variant 371 sequence data have been submitted to MLST database (http://bigsdb.pasteur.fr/)
